# Supplementary material for: Dynamic transcriptomic profiles of zebrafish gills in response to zinc supplementation
Source: BMC Genomics. 2010 Oct 11;11:553. doi: 10.1186/1471-2164-11-553 (PMC3091702; doi:10.1186/1471-2164-11-553)
Supplement: Additional file 2 — Interactive Direct Interaction Network representing the molecular interactions between zinc, copper, iron, calcium and proteins encoded by transcripts changed by zinc supplementation. Mini web-site containing index.html and hyperlinked pages in subdirectory describing a Direct Interaction Network automatically generated based on curated interactions contained within the proprietary PathwayArchitect database. Ovals represent proteins and the circles symbolize metal ions. Objects are coloured by their abundance in zebrafish at the time-point they were significantly different from the control is a scale from -4 fold (dark green) to +4 fold (dark red). Where significant differences were found at more than one time-point, the colour overlay shows expression at the first instance. Dark blue squares denote 'binding', and light blue squares 'expression'; green squares stand for 'regulation', green diamonds for 'metabolism', and green circles for 'promoter binding'. Arrow heads indicate directionality of the interaction where annotated. All nodes and edges can be further interrogated by selecting the relative area of the image. [file 1471-2164-11-553-S2.zip › PathwayArchitect Zn xs DIN/612730.html]

# REGULATION:

|  |  |
| --- | --- |
| Type | REGULATION |
| Effect | Negative |


---

|  |  |
| --- | --- |
| Score | 0 |


---

|  |  |
| --- | --- |
| Reference Count | 8 |


---

|  |  |
| --- | --- |
| Mechanism | Unknown |


---

|  |  |
| --- | --- |
| Reference:0 || Sentence | "An intraluminal interaction may occur, because a fivefold excess of zinc to iron (15 mgZn/3 mg Fe) reduced iron absorption by 56% when given in a water solution but not when given with a hamburger meal." |
| PMID | 2058577 |
| Year | 1991 |
| Species | Human |
| Journal | Am J Clin Nutr |
| RefScore | 2 |
| Source | PArchNLP |
  |
|  |  |


---

|  |  |
| --- | --- |
 Reference:1 || Sentence | "These results support the possibility that zinc deficiency may alter tissue availability of other nutrients such as vitamin A or iron through its effect on transport proteins." |
| PMID | 6792896 |
| Year | 1981 |
| Species | Human |
| Journal | Am J Clin Nutr |
| RefScore | 1 |
| Source | PArchNLP |
  ||  |  |


---

|  |  |
| --- | --- |
 Reference:2 || Sentence | "Zn deficiency in TM may cause hyperzincuria, high ferritin levels, hepatic iron load, hepatic dysfunction." |
| PMID | 11787992 |
| Year | 2001 |
| Species | Human |
| Journal | J Trace Elem Med Biol |
| RefScore | 0 |
| Source | PArchNLP |
  ||  |  |


---

|  |  |
| --- | --- |
 Reference:3 || Sentence | "An inverse correlation (r2: 0.96) was observed between the capacity of zinc to prevent iron binding to the membrane and the inhibitory effect of zinc on Fe2+-initiated lipid oxidation." |
| PMID | 11440839 |
| Year | 2001 |
| Journal | Free Radic Biol Med |
| RefScore | 2 |
| Source | PArchNLP |
  ||  |  |


---

|  |  |
| --- | --- |
 Reference:4 || Sentence | In addition, Zn deficiency caused changes in the levels of Zn and other trace elements such as Ca, Al, S, Fe, and Cl and vitamin C. |
| Year | 2004 |
| PMID | 15103203 |
| Species | Rat |
| Journal | Ophthalmic Res |
| RefScore | 1 |
| Source | PArchNLP |
  ||  |  |


---

|  |  |
| --- | --- |
 Reference:5 || Sentence | Zinc depletion produced mild-moderate iron deficiency in addition to zinc deficiency, which could be corrected by repletion with iron and zinc. |
| PMID | 16198227 |
| Year | 2005 |
| Species | Rat |
| Journal | Free Radic Biol Med |
| RefScore | 3 |
| Source | PArchNLP |
  ||  |  |


---

|  |  |
| --- | --- |
 Reference:6 || Sentence | Negative effects of Zn supplementation on Fe absorption were associated with increased small intestine Fe retention, decreased hephaestin, and increased FPN expression. |
| PMID | 16614402 |
| Year | 2006 |
| Species | Rat |
| Journal | J Nutr |
| RefScore | 1 |
| Source | PArchNLP |
  ||  |  |


---

|  |  |
| --- | --- |
 Reference:7 || Sentence | Iron uptake by HEK and Sf9 cells expressing Zip14 was inhibited by zinc. |
| PMID | 16950869 |
| Year | 2006 |
| Species | Mouse |
|  | Human |
| Journal | Proc Natl Acad Sci U S A |
| RefScore | 2 |
| Source | PArchNLP |
  |


---

|  |  |
| --- | --- |
